# *Helicobacter pylori* Eradication Therapy for Functional Dyspepsia: A Meta-Analysis by Region and *H. pylori* Prevalence

**DOI:** 10.3390/jcm8091324

**Published:** 2019-08-28

**Authors:** Seung Joo Kang, Boram Park, Cheol Min Shin

**Affiliations:** 1 Department of Internal Medicine, Seoul National University Hospital Healthcare System Gangnam Center, Seoul 06236, Korea; 2 Department of Public Health Science, Seoul National University, Seoul 08826, Korea; 3 Department of Internal Medicine, Seoul National University Bundang Hospital, Seongnam 13620, Korea

**Keywords:** *Helicobacter pylori*, eradication, functional dyspepsia, prevalence

## Abstract

Background: Previous studies on the effect of *Helicobacter pylori* eradication on functional dyspepsia (FD) are conflicting. We performed a comprehensive meta-analysis on this issue according to region and prevalence of *H. pylori*. Methods: Randomized controlled trials (RCTs) evaluating the effect of eradication of *H. pylori* on functional dyspepsia up to December 2018 were searched through PubMed, EMBASE, and the Cochrane Library. Subgroup analyses by the outcome measure, region, and prevalence of *H. pylori* were performed. All data were analyzed with Review Manager 5.3. Results: Eighteen RCTs were included in our meta-analysis. Overall, the *H. pylori* eradication group showed significant improvement of symptoms compared with the control group (risk ratio (RR) = 1.18; 95% confidence interval (CI): 1.07–1.30, *p* < 0.01). There was moderate heterogeneity among studies (*I*^2^ = 34%) and the number needed to treat (NNT) was 15.0. *Helicobacter pylori* eradication improved dyspeptic symptoms both in low (<50%) and high (≥50%) *H. pylori* prevalence regions (RR = 1.21 and 1.17; 95% CI: 1.02–1.44 and 1.06–1.29, *I*^2^ = 49% and 5%, respectively.) In the analysis of studies from Asia, however, the effect of eradication on improvement of dyspepsia was not significant (RR = 1.14; 95% CI: 0.99–1.33, *p* = 0.08, *I*^2^ = 37%). Conclusion: Overall, *H. pylori* eradication provides significant improvement of symptoms in functional dyspepsia patients regardless of *H. pylori* prevalence. However, in the analysis of studies from Asia, the eradication did not significantly improve dyspeptic symptoms. In this region, eradication for dyspepsia can be individualized.

## 1. Introduction

Functional dyspepsia (FD) is characterized by bothersome epigastric pain or burning, postprandial fullness, or early satiation without evidence of structural disease [1]. Functional dyspepsia is a very common disease with a prevalence of about 10% of general population and the socioeconomic burden of disease is substantial due to the frequent visits to healthcare facilities and repeated medications and investigations [2]. The pathogenesis of FD includes diverse mechanisms such as diet factor, psychological distress, a disturbance of gastric physiology, duodenal inflammation, and infectious causes represented by *Helicobacter pylori* [3]. This infection was estimated to be about 2.3 fold in patients with dyspepsia compared with normal controls and *H. pylori* was found in about half of the patients with dyspepsia [4]. Therefore, there have been many studies on whether *H. pylori* eradication therapy is effective in relieving the symptoms of dyspepsia. However, the effect of eradication therapy in FD patients was inconsistent in previously published randomized controlled trials (RCTs). The previous meta-analysis that analyzed the long-term effects over 12 months showed that eradication therapy was effective in symptom improvement, but heterogeneity among studies was significant [5]. Causes of heterogeneity have not been clearly elucidated. Thus, we conducted a meta-analysis including recent randomized trials and performed subgroup analyses according to the complete responsiveness, geographical region, and the prevalence of *H. pylori*.

## 2. Materials and Methods

### 2.1. Search Strategy and Study Selection

We implemented current PRISMA (Preferred Reporting Items for Systemic Reviews and Meta-Analyses) guideline for this meta-analysis [6]. The PubMed, EMBASE, and Cochrane Library were searched for published studies in English from 1 January 1997 to 31 December 2018. The main search methodology was the combinations of the following keywords: (“*Helicobacter pylori*” OR “Helicobacter” OR “*H. pylori*”), (“eradication” OR “therapy” OR “treatment” OR “antibiotics” OR “proton pump inhibitors”), and (“dyspepsia” OR “functional dyspepsia” OR “non-ulcer dyspepsia” OR “functional GI disorder”). Two investigators (S.J.K. and C.M.S.) searched and selected the articles independently according to the inclusion and exclusion criteria below. Studies were considered eligible if they met the following criteria: (1) study design: RCTs; (2) study population: adult patients with investigated dyspepsia with endoscopy. Dyspepsia was defined by pain or discomfort centered in the upper abdomen or Rome criteria; (3) intervention: *H. pylori* eradication with a triple regimen containing proton pump inhibitors (PPIs) or Histamine-2 blocker with at least more than two kinds of antibiotics; (4) outcome: changes in dyspeptic symptoms and/or safety related with eradication therapy; (5) follow-up of patients for more than 6 months. Case reports, observational studies, review articles, and published only in abstract forms were excluded from the meta-analysis. This study was a systemic review and meta-analysis and was exempted from requiring the approval of the Institutional Review Board (IRB) because it posed nearly no harm to humans.

### 2.2. Data Extraction and Quality Assessment

Two investigators (S.J.K. and C.M.S.) reviewed and separately extracted data from each paper meeting the inclusion criteria. Details of authors, year of publication, study design, *H. pylori* diagnosis methods, regimens for *H. pylori* eradication, number of patients in control and intervention group, and number of adverse events were extracted from selected articles. Both investigators assessed the quality of studies according to the Cochrane collaboration’s tool for the risk of bias, which contains random sequence generation, allocation concealment, degree of blindness, incomplete outcome data, selective outcome reporting, and other biases [7]. If there were any disagreements among the two investigators, a consensus was drawn after a full discussion.

### 2.3. Study Endpoints and Subgroup Analysis

The primary outcome of this study was the pooled risk ratio (RR) of the resolution or presence of only minimal dyspeptic symptoms after treatment with a 95% confidence interval (CI). The secondary outcomes were pooled RRs of resolution or presence of only mild dyspeptic symptoms in subgroups according to geographical region and *H. pylori* prevalence and pooled RRs of adverse events in both groups. The prevalence of *H. pylori* in each country was based on the data from a systemic review and meta-analysis written by Hooi et al. [8]. They searched all reports of *H. pylori* prevalence from 1 January 1970 to 1 January 2016 in MEDLINE and EMBASE and calculated pooled *H. pylori* prevalence rates and 95% confidence intervals for each country.

### 2.4. Statistical Analysis

We used Review Manager 5.3 (Copenhagen: The Nordic Cochrane Center, The Cochrane Collaboration, 2014) to perform the meta-analysis. Dichotomous outcomes were calculated with RRs and a 95% CI. A random-effects model was applied using the Mantel–Haenszel test for binary outcomes. Subgroup analysis was performed to evaluate the effect of *H. pylori* eradication therapy according to regions and the prevalence of *H. pylori*. The prevalence of *H. pylori* in each country was identified from the study by Hooi et al. [8]. We examined heterogeneity among studies using χ^2^ and *I^2^* statistics. If substantial heterogeneity was identified, the possible clinical causes were assessed, and sensitivity analyses were performed by subgroup as described before. Publication bias was assessed by analyzing the asymmetry of a funnel plot.

## 3. Results

### 3.1. Results of Literature Search and Description of Included Studies

**Figure 1:**
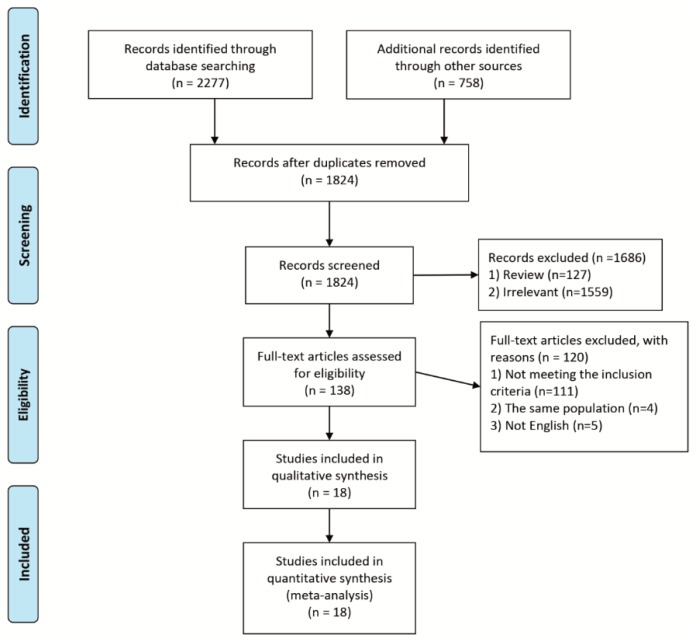
Study flow diagram.

The flowchart of the selection for the studies is shown in Figure 1. From a thorough literature search, a total of 3035 studies were identified from three databases. After removing the duplicates (*n* = 1211), two reviewers screened the potentially relevant studies (*n* = 1824) from titles and abstract independently. Review articles (*n* = 127) and irrelevant articles (*n* = 1559) were excluded from screening and full texts were reviewed for the 138 eligible articles. Finally, 18 RCTs [9,10,11,12,13,14,15,16,17,18,19,20,21,22,23,24,25,26] with a total of 4774 subjects which met the inclusion criteria were included in the meta-analysis. Table 1 shows the main characteristics of the studies that met the inclusion criteria. The criteria and duration for dyspepsia, methods for severity of dyspepsia and quality of life, and definition of treatment success are summarized in Table 2. The risk of bias for each RCT is shown in Figures S1 and S2. Because we included only articles on functional dyspepsia patients with endoscopically excluded diseases, a study by Chiba et al. [27], which randomized uninvestigated dyspepsia patients and was included in previous studies with a meta-analysis [5,28], was excluded from this analysis. In Table S1, the list of excluded RCTs that were previously included in other meta-analyses and the reasons for their exclusion are identified.

### 3.2. Effect of *H. pylori* Eradication Therapy on Symptom Improvement

**Figure 2:**
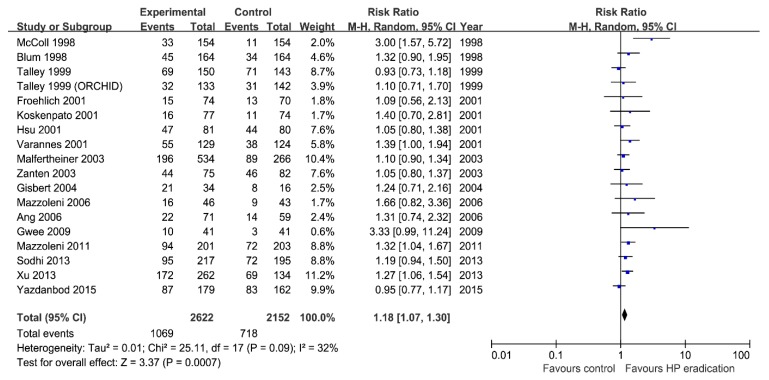
Forest plot for the effect of *Helicobacter pylori* eradication on the improvement of symptoms in patients with functional dyspepsia by random-effects analysis. CI: confidence interval.

**Figure 3:**
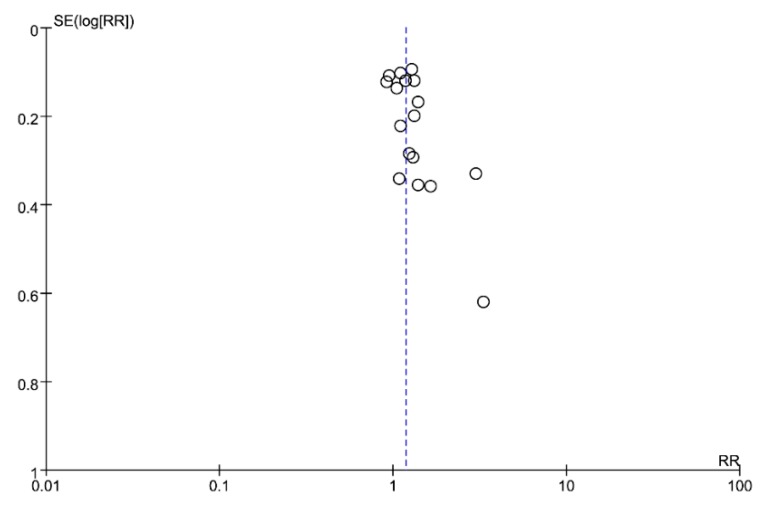
Funnel plot of included studies for potential publication bias.

Meta-analysis of 18 RCTs showed that overall 1069/2622 (40.8%) patients in the *H. pylori* eradication therapy group had improvement of dyspeptic symptoms compared with 718/2152 (33.4%) in the control group (Figure 2). Overall effect of *H. pylori* eradication therapy on dyspepsia symptoms improvement was statistically significant (RR = 1.18; 95% CI: 1.07–1.30, *p* < 0.01). Heterogeneity among included RCTs was moderate (*I*^2^ = 32%, *p* = 0.09). The number needed to treat (NNT) was 15.0 (95% CI: 10.7–25.0). The funnel plot of the 18 included studies showed mild asymmetry around the central line (Figure 3). We performed subgroup analysis according to the degree of responsiveness and complete resolution of symptom and improvement of symptom (Figure S3). When we performed subgroup analysis for studies that adopted complete resolution of symptom as an endpoint, *H. pylori* eradication showed borderline effect (RR = 1.14; 95% CI: 0.99–1.31, *p* = 0.05). Heterogeneity among studies were moderate (*I*^2^ = 46%, *p* = 0.05). For studies which had mild improvement as an endpoint, the effect of eradication was significant (RR = 1.26; 95% CI: 1.13–1.42, *p* < 0.01). Heterogeneity among studies was negligible (*I*^2^ = 0%, *p* = 0.80).

### 3.3. Subgroup Analysis According to *H. pylori* Prevalence and Geographical Region

**Figure 4:**
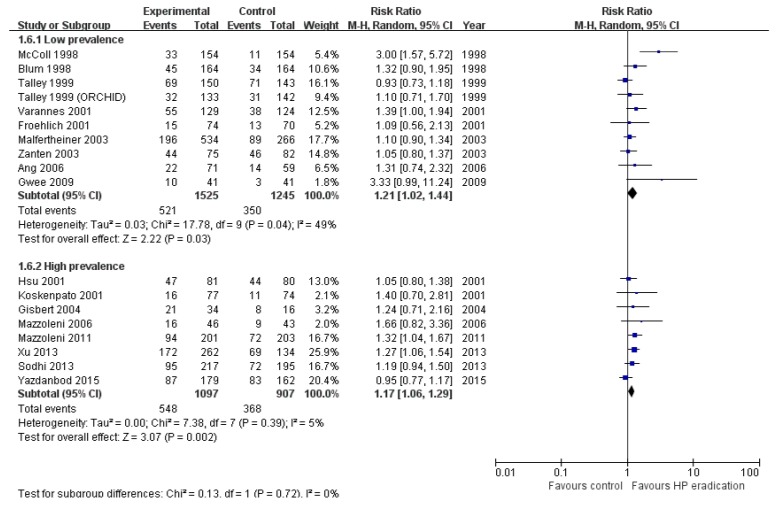
Subgroup analysis by prevalence of *H. pylori.* 1.6.1. Low prevalence: studies from countries with *H. pylori* prevalence < 50%. 1.6.2. High prevalence: studies from countries with *H. pylori* prevalence ≥ 50% (*H. pylori* prevalence was estimated from study by Hooi et al. [8]).

We performed subgroup analysis according to the *H. pylori* prevalence. According to the systemic review and meta-analysis of global prevalence of *H. pylori* infection by Hooi et al. [8], the mean prevalence of *H. pylori* infection except the African region was around 50%. We divided the studies into those from high prevalence area (≥50%) and those from low prevalence area (<50%) (Figure 4). In low prevalence areas, eradication was effective for dyspeptic symptoms (RR = 1.21; 95% CI: 1.02–1.44, *p* = 0.03). However, the heterogeneity was significant (*I*^2^ = 49%, *p* = 0.04). In high prevalence areas, eradication therapy showed a relieving effect on dyspepsia (RR = 1.17; 95% CI: 1.06–1.29, *p* < 0.01) and heterogeneity among studies were very low (*I*^2^ = 5%, *p* = 0.04).

**Figure 5:**
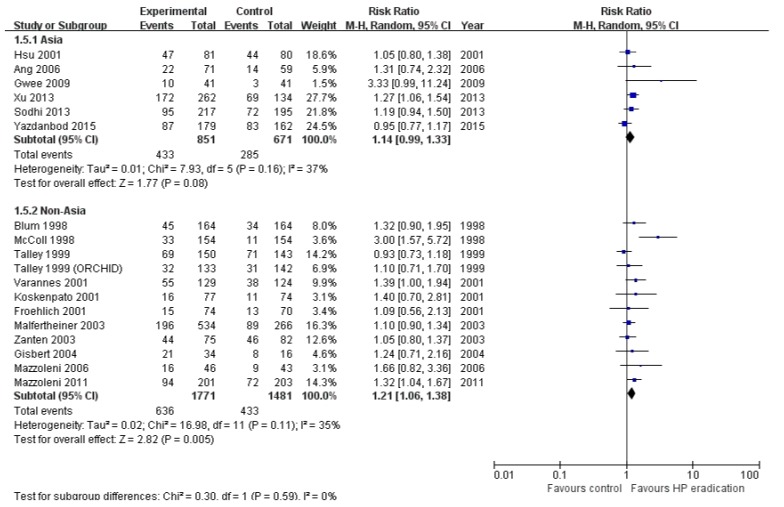
Subgroup analysis by geographical region. 1.5.1. studies from Asia, 1.5.2. studies from outside Asia.

To investigate the difference in the effects of *H. pylori* eradication by region, we performed subgroup analysis according to geographical region (Figure 5). Analysis of six studies from Asia showed that *H. pylori* eradication did not significantly improve the dyspeptic symptoms (RR = 1.14; 95% CI: 0.99–1.33, *p* = 0.08) and heterogeneity among studies were moderate (*I*^2^ = 37%, *p* = 0.16). Analysis of the studies from outside Asia showed that *H. pylori* eradication was effective in improving the symptoms of dyspepsia (RR = 1.21; 95% CI: 1.06–1.38, *p* < 0.01) and heterogeneity was also moderate (*I*^2^ = 35%, *p* = 0.16). Most regions in Asia showed a high prevalence of *H. pylori* but the prevalence was low in areas such Singapore. Therefore, we conducted a subgroup analysis according to the high- and low-prevalence of *H. pylori* in Asia and other regions; the results are shown in the Figure S4. The effect of eradication on dyspepsia was significant in low-prevalence region (RR = 1.21; 95% CI: 1.02–1.44, *p* = 0.03) and high-prevalence region outside Asia (RR = 1.34; 95% CI: 1.10–1.63, *p* < 0.01). However, the effect was attenuated in high-prevalence areas in Asia (RR = 1.12; 95% CI: 0.97–1.28, *p* = 0.12).

### 3.4. Adverse Events of *H. pylori* Eradication Therapy

**Figure 6:**
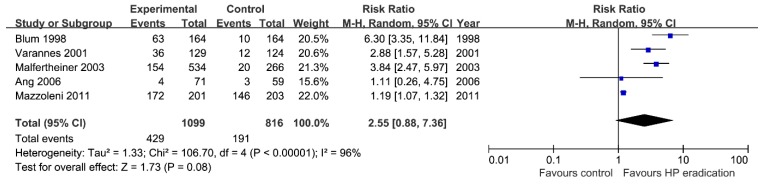
Forest plot of adverse effects associated with *Helicobacter* eradication therapy.

Five studies identified adverse effects associated with treatment. The frequency of side effects was not significantly higher in the group receiving *H. pylori* eradication therapy than in the control group (RR = 2.55; 95% CI: 0.88–7.36, *p* = 0.08) (Figure 6). Most adverse event was mild and included diarrhea, abnormal tastes, and malaise. Serious adverse event was very rare according to studies. However, heterogeneity among studies was very high (*I*^2^ = 96%, *p* < 0.01).

## 4. Discussion

In this meta-analysis, *H. pylori* eradication therapy showed a statistically significant long-term effect, although the effect size was small, in patients with functional dyspepsia. However, the heterogeneity among studies was moderate and the NNT was 15.0 (95% CI: 10.7–25.0), which might be acceptable only if the clinical situation is not urgent and adverse effects are mild [29]. Subgroup analysis according to the degree of response did not show a significant effect on complete resolution of symptom but showed significant effect on some degree of improvement. *H. pylori* eradication was effective in improving the dyspeptic symptom regardless of the prevalence of *H. pylori* infection.

The pathophysiology of dyspepsia includes an altered gut-brain axis which causes abnormal central pain processing and results in gastric hypersensitivity. A disturbance of gastric physiologies, such as delayed gastric emptying and impaired accommodation, also plays an important role in the pathogenesis of functional dyspepsia. *H. pylori* infection is thought to be one of the important causes of functional dyspepsia. *H. pylori* induces changes in gastric acid secretion by altering gastrin and somatostatin production [30]. Excessively increased gastric acid secretion by any causes can cause dyspeptic symptoms in experiments with healthy volunteers [31]. Based on these grounds, many RCTs have been conducted to find out if *H. pylori* eradication therapy is helpful in the treatment of dyspepsia. As shown in the results of meta-analysis, collectively, *H. pylori* eradication was effective in relieving the dyspeptic symptom. However, the estimated NNT was 15 and heterogeneity among studies was moderate. In order to find the cause of heterogeneity, we performed subgroup analysis according to the degree of response. The effect of eradication on complete resolution of symptom was not statistically significant (*p* = 0.07), while the effect on mild improvement of symptom was significant (*p* < 0.01).

*H. pylori* infection exerts diverse effects on gastric acid secretion which depends on the pattern of gastritis caused by the infection [32]. If *H. pylori* causes an antral predominant non-atrophic gastritis, gastric acid secretion is increased, leading to duodenal ulcer disease [33]. In patients with atrophic gastritis by *H. pylori*, gastric acid secretion decreases and gastric cancer risk increases [34]. The degree of gastric acid secretion affects the area of gastritis caused by *H. pylori* [35]. The degree of gastritis and gastric acid secretion by *H. pylori* interacts with each other, which causes different responses to treatment of *H. pylori* eradication. This can be one of the causes of heterogeneous results of each randomized study.

Inflammation is deeply involved in the pathogenesis of functional dyspepsia. Increased numbers of mast cells and augmented expression of histamine, serotonin, and tryptase in gastric mucosal biopsy samples were observed in both patients with post-infectious functional dyspepsia or unspecified functional dyspepsia compared with healthy controls [36]. Immune activation in duodenum is one of the important pathophysiology of functional dyspepsia. The numbers of eosinophils and mast cells are increased in duodenal bulb and second portion in patients with functional dyspepsia [37]. Low-grade inflammation affects the barrier function of the stomach and duodenum. An impaired duodenal barrier function can lead to acid hypersensitivity in functional dyspepsia by facilitating the passage of H^+^ ions through the epithelium, which can subsequently reach acid-sensing receptors located on visceral afferent nerve endings [36]. In one study that reviewed 51 reports which investigated histological changes after the eradication of *H. pylori*, the degree of activity of gastritis and inflammation significantly improved in nearly all reports, whereas gastric atrophy improved in about half of reports and only 18% of reports showed significant improvement in intestinal metaplasia [38]. Whether the improvement of gastric inflammation followed by eradication therapy is related with symptom relief is an important issue. In the study by Optimal Regimen Cures *Helicobacter* Induced Dyspepsia (ORCHID) Study Group, in line with previous reports, 81% of patients in the treatment group had no or mild chronic gastritis at 12 months after eradication compared with 13% in the placebo group [12]. However, overall treatment success which was defined as minimal or no dyspeptic symptoms was 24% in the treatment group and 31% in the placebo group. There was no significant difference among the two groups. As a secondary analysis, they divided patients according to the follow-up period into those with a chronic gastritis score 0 or 1 and those with a score of 2 or 3 regardless of treatment. At the 12 month follow-up, 32% of patients with no or mild gastritis were considered a treatment success compared with 17% with moderate or severe gastritis (*p* = 0.008). Although this association between healing of chronic gastritis and symptom relief requires further confirmation, it can be interpreted as indirect evidence that improvement of inflammation followed by *H. pylori*.

Another point to note is that the subgroup analysis of studies from Asia did not show statistically significant effects on dyspepsia. Three reasons can be considered for this result. First, six studies were performed in the Asia region. Except for one study by Xu et al. [24], all studies from Asia adopted a strict endpoint for their outcome. It is possible that this has made the efficacy of eradication to be underestimated. Second, the total number of patients in the studies from Asia was 1522, whereas, those of patients in studies outside Asia was 3252. The confidence interval of RR in meta-analysis of Asian studies was wider than that in the meta-analysis of studies outside Asia. Therefore, more studies from Asia are needed to clearly elucidate the efficacy of eradication. In a previous meta-analysis published in 2014, analysis of four RCTs from Asia showed a significant effect of eradication on dyspepsia [5]. Later, however, two large scale RCTs came out in Asia, which showed all negative results [25,26]. Combined meta-analysis of six studies gave an insignificant result. This inconsistency between meta-analysis seems to be due to the lack of sufficient number of RCTs from Asia, which is why further studies on this issue are needed. Finally, the difference in eating habits might have affected. It is well known that Asians have a high consumption of spicy foods and that excessive consumption of spicy foods is associated with development of dyspepsia [39]. The effect of eradication may also be underestimated compared with non-Asian studies due to the differences in food culture.

In guidelines on functional dyspepsia made in the Asia-Pacific region, Japan, and the United States and Canada, eradication is strongly recommended as a primary treatment in patients with *H. pylori*-positive dyspepsia [40,41,42]. Our findings support the recommendations from the above guidelines as the *H. pylori* eradication group showed significant improvement of symptoms, although the difference was small. In subgroup analysis studies from Asia, the effect was not significant. However, as discussed above, the interpretation of studies from Asia requires caution. Furthermore, as Asia has a high prevalence of *H. pylori* and a high incidence of gastric cancer, recommendation for eradication is reasonable considering the additional benefit of reducing the incidence of ulcers and gastric cancer [40]. It is important to note that these studies are based on patients with investigated dyspepsia. The results from this meta-analysis do not apply to the patients with uninvestigated dyspepsia. Recent guidelines recommend the test-and-treat strategy for uninvestigated dyspepsia patients under the age of 60 [42]. However, decisions on endoscopy require comprehensive consideration of the *H. pylori* prevalence in the area, the accessibility to the endoscopy, and alarm symptoms of patients. In our study, eradication for investigated *H. pylori*-positive dyspepsia was effective both in low- and high-prevalence areas. In low-prevalence areas, indiscriminate non-invasive testing can produce a large number of negative results, and this should be considered for therapeutic approaches. Indeed, German guidelines do not recommend general use of the test-and-treat strategy [43]. In the Kyoto Consensus, a group of patients with *H. pylori*-positive dyspepsia who showed sustained symptom relief from 6 months or longer after eradication was defined as *H. pylori*-associated dyspepsia as a separate clinical entity [44]. Our meta-analysis showed that about 40% of *H. pylori*-positive dyspepsia patients have symptomatic relief after 12 months after eradication and these can be classified as *H. pylori*-associated dyspepsia. This approach emphasizes the diagnosis and treatment of *H. pylori* over other treatments and is considered a reasonable approach in areas with a high prevalence of *H. pylori* and high burden of disease caused by *H. pylori*.

The prevalence of dyspepsia diagnosed using the Rome III criteria is known to be 5.3–20.4% of the general population and *H. pylori* infection among dyspeptic patients is estimated to be up to 70% of dyspeptic patients from a population-based study, although it showed regional differences [45]. For a clinical application of *H. pylori* eradication in dyspepsia, we should consider benefit and risk of mass eradication of *H. pylori*, especially in high *H. pylori* prevalence area. A systemic review which investigated the effect of *H. pylori* eradication on the incidence of gastric cancer in asymptomatic individuals showed that individuals with eradication of *H. pylori* have a lower incidence of gastric cancer than those who did not receive eradication therapy (pooled incidence rate ratio = 0.53; 95% CI: 0.44–0.64) [46]. Baseline gastric cancer incidence modified the benefit of *H. pylori* eradication. That is, reduction of cancer incidence increased in intermediate and high gastric incidence area. On the other hand, the increase in antibiotic resistance needs to be considered. Increasing trends in the antibiotic resistance of *H. pylori* has been consistently reported in many studies from various regions [47]. A resistance to clarithromycin and levofloxacin is mostly due to the use of these drugs for infectious diseases other than *H. pylori* infection.

It is worth mentioning the subgroup analysis according to the outcome. Since functional dyspepsia lacks effective biomarkers, it is not yet clear which outcome variable is good for evaluating the effectiveness of the treatment. Currently available outcome measures are heterogeneous because functional dyspepsia is a complex of various symptoms [48]. We analyzed the outcome by dividing it into “complete resolution” and “mild improvement”. “Complete resolution” is no residual symptom or minimal symptom with a Likert score of 1. “Mild improvement” is when there is 50% or more reduction in the initial symptom score. Because functional dyspepsia has chronic and wax-and-wane nature, both endpoints seem to be clinically meaningful indicators. However, 50.7% (418/824) of patients were effective with “mild improvement” as an endpoint and only 36.2% (651/1798) of patients with a “complete resolution” endpoint. Therefore, although “complete resolution” is considered to be a stricter outcome, both are indicators of clinical usefulness and can be used according to the research purpose.

The limitations of this study need to be mentioned. First, the eradication rate of included studies ranged from 62.1% to 91.3%. However, many studies did not provide data about symptom relief in eradicated patients and we were not able to assess the improvement of symptoms among the successfully eradicated patients. Second, several clinical parameters, such as subtypes of dyspepsia, presence of atrophic gastritis or intestinal metaplasia, and age, are thought to influence the efficacy of the eradication therapy, but only a few studies have analyzed these factors. Therefore, further research for which subgroup eradication therapy will be particularly effective should be performed. Third, most studies were done in hospital settings. That is, there is the possibility that the composition of the patient groups were different from those seen in primary care settings. Finally, the follow-up period of all studies was 12 months, so it was difficult to conclude with these results on the long-term effects on dyspepsia of more than 1 year.

## 5. Conclusions

In conclusion, *H. pylori* eradication therapy provides statistically significant long-term symptom improvement in functional dyspepsia patients regardless of *H. pylori* prevalence. However, the NNT was 15 and in the analysis of studies from Asia, the eradication showed no significant improvement. Therefore, *H. pylori* eradication therapy for functional dyspepsia requires an individualized approach in the Asia region.

## Figures and Tables

**Table 1:** The characteristics of the studies included in the meta-analysis.

Studies	Country	*H. pylori* Prevalence	Arms (Regimens)	Number of Patients	Mean or Median Age	Eradication Rate (%)	Follow-Up	Adverse Event
McColl, 1998 [9]	UK	35.5%	Omeprazole	160	42.0 ± 12	85%	12 Mo	N/A
			Amoxicillin					
			Metronidazole					
			Omeprazole	158	42.2 ± 13	12%	12 Mo	N/A
Blum, 1998 [10]	Austria, Canada, Germany, Iceland, Ireland, Sweden, South Africa	41.6%	Omeprazole	164	47	79%	12 Mo	7%
			Amoxicillin					
			Clarithromycin					
			Omeprazole	164	47	2%	12 Mo	1%
Talley, 1999 [11]	US	35.6%	Omeprazole	150	46.3	90%	12 Mo	N/A
			Amoxicillin					
			Clarithromycin					
			Placebo	143	46.5	2%	12 Mo	N/A
Talley, 1999 (ORCHID) [12]	Australia, New Zealand, and Europe	28.0%	Omeprazole	133	51	85%	12 Mo	N/A
			Amoxicillin					
			Clarithromycin					
			Placebo	142	47	4%	12 Mo	N/A
Varannes, 2001 [13]	France	46.9%	Ranitidine	129	50 ± 16	69%	12 Mo	28%
			Amoxicillin					
			Clarithromycin					
			Placebo	124	52 ± 14	18%	12 Mo	10%
Koskenpato, 2001 [14]	Finland	56.8%	Omeprazole	77	51.5 ± 9.5	82%	12 Mo	N/A
			Amoxicillin					
			Metronidazole					
			Omeprazole	74	51.8 ± 11.8	1%	12 Mo	N/A
Froehlich, 2001 [15]	Switzerland	18.9%	Lansoprazole	92	43.6 ± 12.4	75%	12 Mo	N/A
			Amoxicillin					
			Clarithromycin					
			Lansoprazole	88	45.6 ± 14.2	4%	12 Mo	N/A
Hsu, 2001 [16]	Taiwan	53.9%	Lansoprazole	81	50.3 ± 15.1	78%	12 Mo	N/A
			Metronidazole					
			Tetracycline					
			Lansoprazole	80	51.6 ± 16.4	0%	12 Mo	N/A
Malfertheiner, 2003 [17]	Germany	35.3%	Lansoprazole	534 (270 (30)/264 (15))	46.1 ± 12.8 (30)	65.6% (30)	12 Mo	7%
			Amoxicillin		46.9 ± 12.0 (15)	62.1% (15)		5%
			Clarithromycin					
			Lansoprazole	133	45.5 ± 12.6	4.5%	12 Mo	6%
Zanten, 2003 [18]	Canada	38.0%	Lansoprazole	75	47 ± 13	82%	12 Mo	N/A
			Amoxicillin					
			Clarithromycin					
			Placebo	82	49 ± 13	6%	12 Mo	N/A
Gisbert, 2004 [19]	Spain	54.9%	Omeprazole	34	42	76%	12 Mo	N/A
			Amoxicillin					
			Clarithromycin					
			Ranitidine	16	41	0%	12 Mo	N/A
Mazzoleni, 2006 [20]	Brazil	71.2%	Lansoprazole	46	43.2 ± 11.9	91.3%	12 Mo	N/A
			Amoxicillin					
			Clarithromycin					
			Lansoprazole	45	39.2 ± 13.8	0%	12 Mo	N/A
Ang, 2006 [21]	Singapore	40.8%	Lansoprazole	71	38.6	73.2%	52 wk	6%
			Amoxicillin					
			Clarithromycin					
			Prokinetic 6 wk	59	38.4	0%	52 wk	5%
Gwee, 2009 [22]	Singapore	40.8%	Omeprazole	41	44.7 ± 11.4	68.3%	12 Mo	N/A
			Clarithromycin					
			Tinidazole					
			Placebo	41	36.1 ± 12.1	4.9%	12 Mo	N/A
Mazzoleni, 2011 [23]	Brazil	71.2%	Omeprazole	201	46.1 ± 12.4	88.6%	12 Mo	93%
			Amoxicillin					
			Clarithromycin					
			Omeprazole	203	46.0 ± 12.2	7.4%	12 Mo	82%
Xu, 2013 [24]	China	55.8%	Triple therapy	138	44.4 ± 10.2	80.5%	52 wk	N/A
					42.6 ± 10.3	71.8%		
			Sequential therapy	124				
			Talcid or Domperidone		40.0 ± 11.6		52 wk	N/A
Sodhi, 2013 [25]	India	63.5%	Omeprazole	259	46 (25–65)	69.9%	12 Mo	N/A
			Amoxicillin					
			Clarithromycin					
			Omeprazole	260	43 (20–68)	5.0%	12 Mo	N/A
Yazdanbod, 2015 [26]	Iran	59.0%	Omeprazole	186	36.8	87.1%	12 Mo	N/A
			Bismuth subcitrate					
			Amoxicillin					
			Clarithromycin					
			Omeprazole	173	36.8	2.9%	12 Mo	N/A

N/A, not available. *H. pylori*, *Helicobacter pylori*; Mo, months; wk, weeks.

**Table 2:** Definition of dyspepsia and symptom assessment.

Studies	Definition of Dyspepsia	Duration of Dyspepsia	Severity of Dyspepsia Assessment	Quality of Life Assessment	Treatment Success	*H. pylori* Test	Post-Eradication Test	Allowance for Medication
McColl, 1998 [9]	Intermittent or persistent pain or discomfort in the upper abdomen, heartburn, nausea, a feeling of postprandial fullness, or any other symptoms thought to be related to the upper GI tract	4 Mo	GDSS	SF-36	A score of 0 or 1 on the GDSS	UBT, CLO, Histology	UBT	The patients could take any medication necessary, including PPI
Blum, 1998 [10]	Dyspeptic symptoms (specifically, pain or discomfort centered in the upper abdomen) that had been present for at least six months	6 Mo	Mean symptom score by Likert score	GSRS, Psychological General Well-Being Index	No symptoms or no more than minimal pain or discomfort (a score of 0 or 1) centered in the upper abdomen during any of the 7 days preceding the 12 month visit	UBT, CLO, Histology	UBT, CLO, Histology	Not specified
Talley, 1999 [11]	Moderate pain of discomfort centered in the upper abdomen as their predominant symptom for a minimum of three days in the week	3 Mo	GSRS	SF-36	No more than mild pain or discomfort centered in the upper abdomen (a score of 0 or 1) during the 7 days before the final visit	UBT	UBT, Histology	Antacid was dispensed at each visit
Talley, 1999 (ORCHID) [12]	Pain or discomfort centered in the upper abdomen	3 Mo	Dyspeptic symptoms using validated Likert scale (0–4)	GSRSPsychological General Well-Being Index	No more than minimal dyspeptic symptoms during any of the 7 days before the 12 month visit	UBT, CLO, Histology	UBT, Histology	Patients could receive treatment for dyspeptic symptoms from their doctor, but all drugs used were recorded
Varannes, 2001 [13]	Intermittent or persistent epigastric pain for at least 3 months with a severity score of 3 or more on a 5-point Likert scale	3 Mo	Likert scale (0–4)	SF-36	A decrease of at least 2 points on the Likert scale between randomization and the 12 month follow-up	CLO, Histology	UBT	Rescue symptomatic medications could be prescribed from day 8 until the end of the study, provided they were not anti-secretory drugs or sucralfate
Koskenpato, 2001 [14]	Dyspeptic symptoms	3 Mo	Numeric scale questionnaire validated in a Finnish population (0–36)	SF-36	Reduction of symptom score ≥ 50%	CLO, Histology, Culture	CLO, Histology, Culture	Omeprazole 20 mg daily for the first 3 months and thereafter placebo during the follow-up
Froehlich, 2001 [15]	Epigastric complaints (symptom score > 7 on a sum score ranging from 5 to 25)	10 days	Validated questionnaire (5–25)	SF-12	Symptom score less than 7	UBT, CLO, Histology	UBT	Not specified
Hsu, 2001 [16]	Pain or discomfort centered in the upper abdomen	3 Mo	Validated questionnaire (0–15)	N/A	Resolution of symptoms, defined as a score below 3	CLO, Histology	UBT, CLO, Histology	Subjects were allowed to take antacids or prokinetics (H2 blocker or PPI were forbidden) but not during the month before each interview
Malfertheiner, 2003 [17]	Patients seeking medical care for dyspeptic symptom	4 wk	Non-ulcer dyspepsia sum score		Non-ulcer dyspepsia sum score of ≤1	CLO	UBT	Not specified
Zanten, 2003 [18]	Rome definition: chronic or frequently recurring epigastric pain which could be associated with other upper GI symptoms	3 Mo	MDSS		Patients were classified as responders if they had a decrease of ≥4 points on the DSS. If patients required H2 blocker, PPI or prokinetics, they were considered as non-responders	UBT, CLO, Histology	UBT	Patients were given aluminum hydroxide-magnesium hydroxide as a rescue antacid
Gisbert, 2004 [19]	Pain or discomfort centered in the upper abdomen	3 Mo	Five-point Likert scale	N/A		CLO, Histology	UBT	No anti-secretory therapy was allowed
Mazzoleni, 2006 [20]	Pain or discomfort centered in the upper abdomen	3 Mo	PADYQ (0–44)	N/A	The proportion of patients presenting a decrease of 50% or more in dyspeptic scores at 12 months compared with the baseline score	CLO, Histology	CLO, Histology	During the study, patients were allowed to use H2 blocker and/or prokinetics to treat dyspeptic symptoms
Ang, 2006 [21]	Pain or discomfort centered in the upper abdomen	3 Mo	GDSS	N/A	The resolution of symptoms, defined as a score of 0 or 1 on the GDSS at 1 year	UBT, CLO	UBT, CLO	Not specified
Gwee, 2009 [22]	Rome II criteria	3 Mo	Dyspepsia score (0–15)	General Health Questionnaire	Symptom resolution was defined as a dyspepsia score of 0 or 1 at the 12 month	UBT	UBT	H2 blocker, antacids, prokinetics were allowed
Mazzoleni, 2011 [23]	Rome III criteria	3 Mo	PADYQ (0–44)	N/A	Proportion of patients with at least a 50% decrease in the dyspeptic symptoms score at 12 months compared with their baseline score.	CLO, Histology	CLO, Histology	H2 blockers and prokinetics were allowed
Xu, 2013 [24]	Rome III criteria	3 Mo	GSRS	N/A	Improvement more than 50% by symptom score	CLO, Histology	UBT	Talcid and domperidone were allowed for control group
Sodhi, 2013 [25]	Rome II criteria	3 Mo	7-points Likert scales	N/A	Patients who reported no more than minimal dyspeptic symptoms (0 or 1) during any of the 7 days before each visit	CLO, Histology	CLO, Histology	
Yazdanbod, 2015 [26]	Rome III criteria	3 Mo	GDSS (0–20)	N/A	Presence of no more than mild pain or discomfort (a score of 0 or 1)	CLO, Histology	UBT	

CLO, *Campylobacter*-like organism test; GDSS, Glasgow dyspepsia severity score; GSRS, gastrointestinal symptom rating scale; H2, histamine 2; *H. pylori, Helicobacter pylori*; Mo, months; MDSS, mean dyspepsia summary score; PADYQ, Porto Alegre dyspeptic symptoms questionnaire; PPI, proton pump inhibitor; SF-36, 36 item medical outcomes study short-form general health survey; UBT, urea breath test; wk, weeks.

## Supplementary Materials

The followings are available online at https://www.mdpi.com/2077-0383/8/9/1324/s1; Table S1: The excluded articles and the reasons for exclusion (articles previously analyzed in other meta-analyses), Figure S1: Summary of risk bias, Figure S2: Risk of bias graph of each study, Figure S3: Subgroup analysis by degree of response for the effect of eradication (A) Strict: Treatment success was defined as no or minimal dyspeptic symptoms, (B) Other: treatment success was defined as decrease or reduction of symptom score compared with baseline. CI: confidence interval; Figure S4: Subgroup analysis by *H. pylori* prevalence and geographical regions (A) Low-prevalence region, (B) High-prevalence region outside Asia, (C) High-prevalence region in Asia. CI: confidence interval.
