# Supplementary material for: Helicobacter pylori Eradication Therapy for Functional Dyspepsia: A Meta-Analysis by Region and H. pylori Prevalence
Source: J Clin Med. 2019 Aug 28;8(9):1324. doi: 10.3390/jcm8091324 (PMC6780123; doi:10.3390/jcm8091324)
Supplement: Supplementary file 1 [file jcm-08-01324-s001.pdf]

Table S1. The excluded articles and the reason of exclusion (Articles previously analyzed in other meta-analysis).

Articles	The Reason of Exclusion	Criteria for this Meta-Analysis
Dhali 1999	Symptom assessed 12 weeks after *H. pylori* eradication	12 months after *H. pylori* eradication
Greenberg, 1999	Changes of dyspeptic symptom scores in each groups were presented.	Number of patients who showed symptom improvements
Goddard, 1999	No control group	Randomized controlled trial
Miwa, 2000	Symptom assessed 12 weeks after *H. pylori* eradication	12 months after *H. pylori* eradication
Chiba, 2002	Patients with uninvestigated dyspepsia	Dyspepsia investigated by endoscopy
Alizadeh-Naeeni, 2002	Symptom assessed 3 months after *H. pylori* eradication	12 months after *H. pylori* eradication
Koelz, 2003	Symptom assessed 6 months after *H. pylori* eradication	12 months after *H. pylori* eradication
Varasa, 2008	Changes of dyspeptic symptom scores in each groups were presented.	Number of patients who showed symptom improvements
Lan, 2011	Symptom assessed 3 months after *H. pylori* eradication	12 months after *H. pylori* eradication

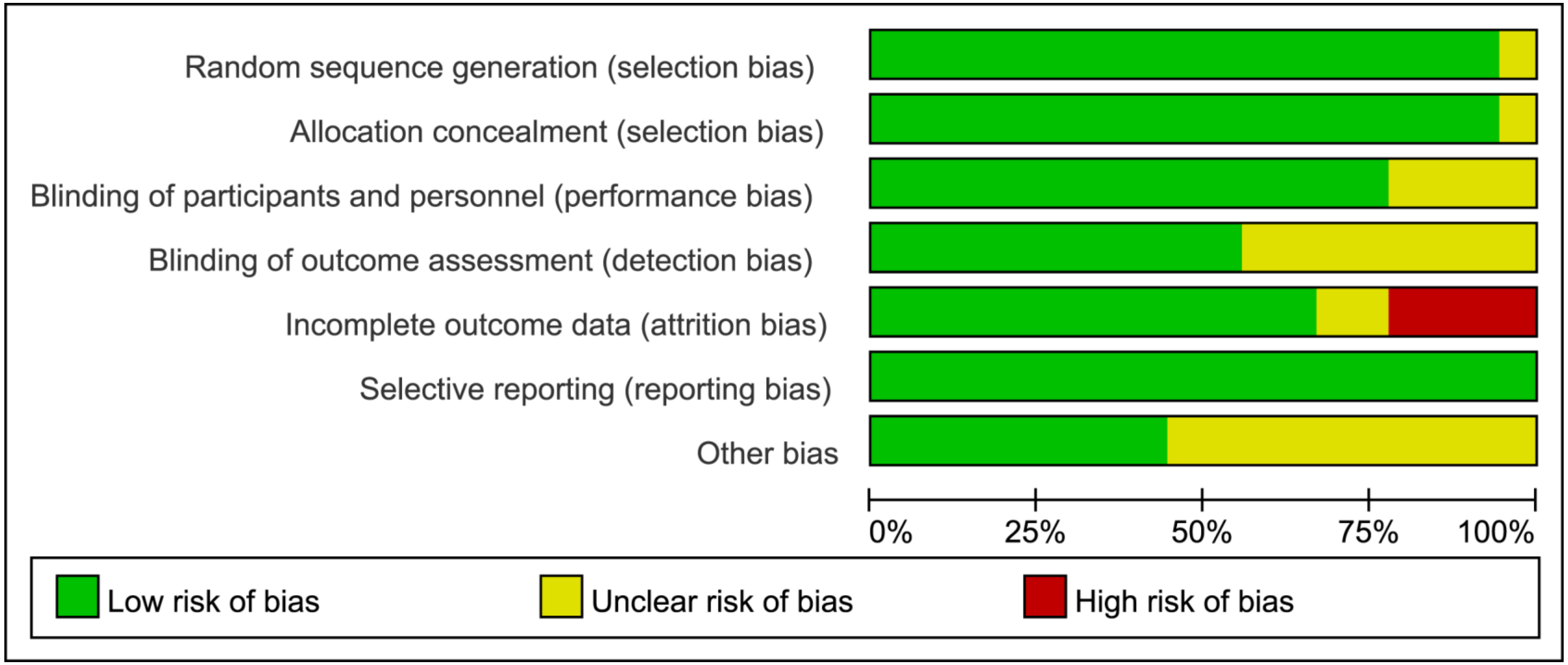

Figure S1. Summary of risk bias.

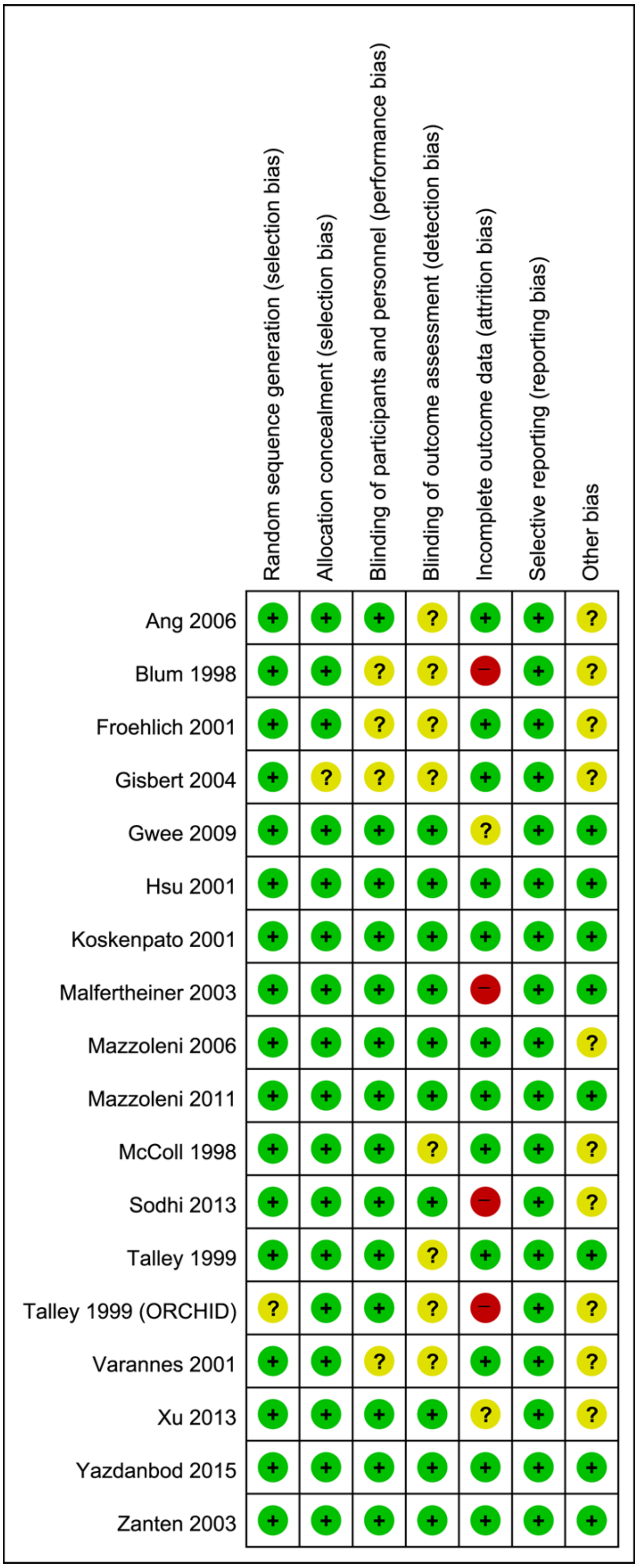

Figure S2. Risk of bias graph of each study.

	Experimental		Control			Risk Ratio	
Study or Subgroup	Events	Total	Events	Total	Weight	M-H, Random, 95% CI	Year
**1.7.1 Strict**							
Blum 1998	45	164	34	164	8.3%	1.32 [0.90, 1.95]	1998
McColl 1998	33	154	11	154	4.0%	3.00 [1.57, 5.72]	1998
Talley 1999	69	150	71	143	13.8%	0.93 [0.73, 1.18]	1999
Talley 1999 (ORCHID)	32	133	31	142	7.2%	1.10 [0.71, 1.70]	1999
Froehlich 2001	15	74	13	70	3.7%	1.09 [0.56, 2.13]	2001
Hsu 2001	47	81	44	80	12.4%	1.05 [0.80, 1.38]	2001
Malfertheiner 2003	196	534	89	266	15.5%	1.10 [0.90, 1.34]	2003
Ang 2006	22	71	14	59	4.8%	1.31 [0.74, 2.32]	2006
Gwee 2009	10	41	3	41	1.3%	3.33 [0.99, 11.24]	2009
Sodhi 2013	95	217	72	195	13.9%	1.19 [0.94, 1.50]	2013
Yazdanbod 2015	87	179	83	162	15.1%	0.95 [0.77, 1.17]	2015
**Subtotal (95% CI)**		**1798**		**1476**	**100.0%**	**1.14 [0.99, 1.31]**	
Total events	651		465				
Heterogeneity: Tau² = 0.02; Chi² = 18.56, df = 10 (P = 0.05); I² = 46%							
Test for overall effect: Z = 1.83 (P = 0.07)							
**1.7.2 Other**							
Koskenpato 2001	16	77	11	74	2.7%	1.40 [0.70, 2.81]	2001
Varannes 2001	55	129	38	124	11.8%	1.39 [1.00, 1.94]	2001
Zanten 2003	44	75	46	82	17.9%	1.05 [0.80, 1.37]	2003
Gisbert 2004	21	34	8	16	4.2%	1.24 [0.71, 2.16]	2004
Mazzoleni 2006	16	46	9	43	2.6%	1.66 [0.82, 3.36]	2006
Mazzoleni 2011	94	201	72	203	23.2%	1.32 [1.04, 1.67]	2011
Xu 2013	172	262	69	134	37.6%	1.27 [1.06, 1.54]	2013
**Subtotal (95% CI)**		**824**		**676**	**100.0%**	**1.26 [1.13, 1.42]**	
Total events	418		253				
Heterogeneity: Tau² = 0.00; Chi² = 3.08, df = 6 (P = 0.80); I² = 0%							
Test for overall effect: Z = 4.01 (P < 0.0001)							
Test for subgroup differences: Chi² = 1.22, df = 1 (P = 0.27), I² = 17.9%							

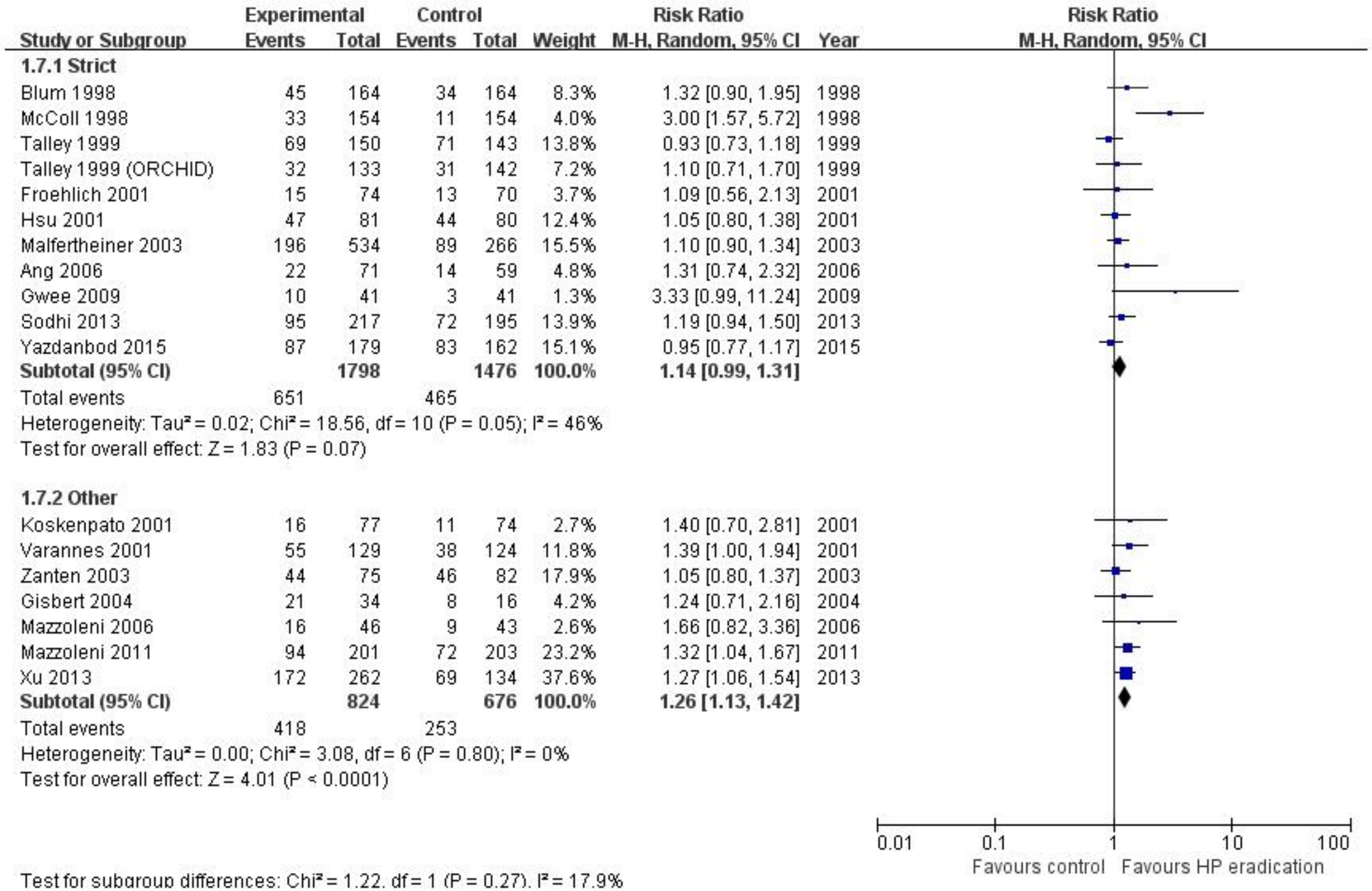

Figure S3. Subgroup analysis by degree of response for the effect of eradication. 1.7.1 Strict: Treatment success was defined as no or minimal dyspeptic symptoms, 1.7.2 Other: treatment success was defined as decrease or reduction of symptom score compared with baseline. CI: confidence interval.

Study or Subgroup	Experimental Events	Experimental Total	Control Events	Control Total	Weight	Risk Ratio M-H, Random, 95% CI	Year
**1.9.1 Low HP prevalence**							
Blum 1998	45	164	34	164	10.6%	1.32 [0.90, 1.95]	1998
McColl 1998	33	154	11	154	5.4%	3.00 [1.57, 5.72]	1998
Talley 1999	69	150	71	143	16.1%	0.93 [0.73, 1.18]	1999
Talley 1999 (ORCHID)	32	133	31	142	9.4%	1.10 [0.71, 1.70]	1999
Varannes 2001	55	129	38	124	12.5%	1.39 [1.00, 1.94]	2001
Froehlich 2001	15	74	13	70	5.2%	1.09 [0.56, 2.13]	2001
Zanten 2003	44	75	46	82	14.8%	1.05 [0.80, 1.37]	2003
Malfertheiner 2003	196	534	89	266	17.7%	1.10 [0.90, 1.34]	2003
Ang 2006	22	71	14	59	6.5%	1.31 [0.74, 2.32]	2006
Gwee 2009	10	41	3	41	1.8%	3.33 [0.99, 11.24]	2009
**Subtotal (95% CI)**		**1525**		**1245**	**100.0%**	**1.21 [1.02, 1.44]**	
Total events	521		350				
Heterogeneity: Tau² = 0.03; Chi² = 17.78, df = 9 (P = 0.04); I² = 49%							
Test for overall effect: Z = 2.22 (P = 0.03)							
**1.9.2 High HP prevalence outside Asia**							
Koskenpato 2001	16	77	11	74	8.2%	1.40 [0.70, 2.81]	2001
Gisbert 2004	21	34	8	16	12.9%	1.24 [0.71, 2.16]	2004
Mazzoleni 2006	16	46	9	43	8.1%	1.66 [0.82, 3.36]	2006
Mazzoleni 2011	94	201	72	203	70.9%	1.32 [1.04, 1.67]	2011
**Subtotal (95% CI)**		**358**		**336**	**100.0%**	**1.34 [1.10, 1.63]**	
Total events	147		100				
Heterogeneity: Tau² = 0.00; Chi² = 0.48, df = 3 (P = 0.92); I² = 0%							
Test for overall effect: Z = 2.86 (P = 0.004)							
**1.9.3 High HP prevalence in Asia**							
Hsu 2001	47	81	44	80	19.1%	1.05 [0.80, 1.38]	2001
Xu 2013	172	262	69	134	31.2%	1.27 [1.06, 1.54]	2013
Sodhi 2013	95	217	72	195	23.1%	1.19 [0.94, 1.50]	2013
Yazdanbod 2015	87	179	83	162	26.6%	0.95 [0.77, 1.17]	2015
**Subtotal (95% CI)**		**739**		**571**	**100.0%**	**1.12 [0.97, 1.28]**	
Total events	401		268				
Heterogeneity: Tau² = 0.01; Chi² = 4.60, df = 3 (P = 0.20); I² = 35%							
Test for overall effect: Z = 1.58 (P = 0.12)							

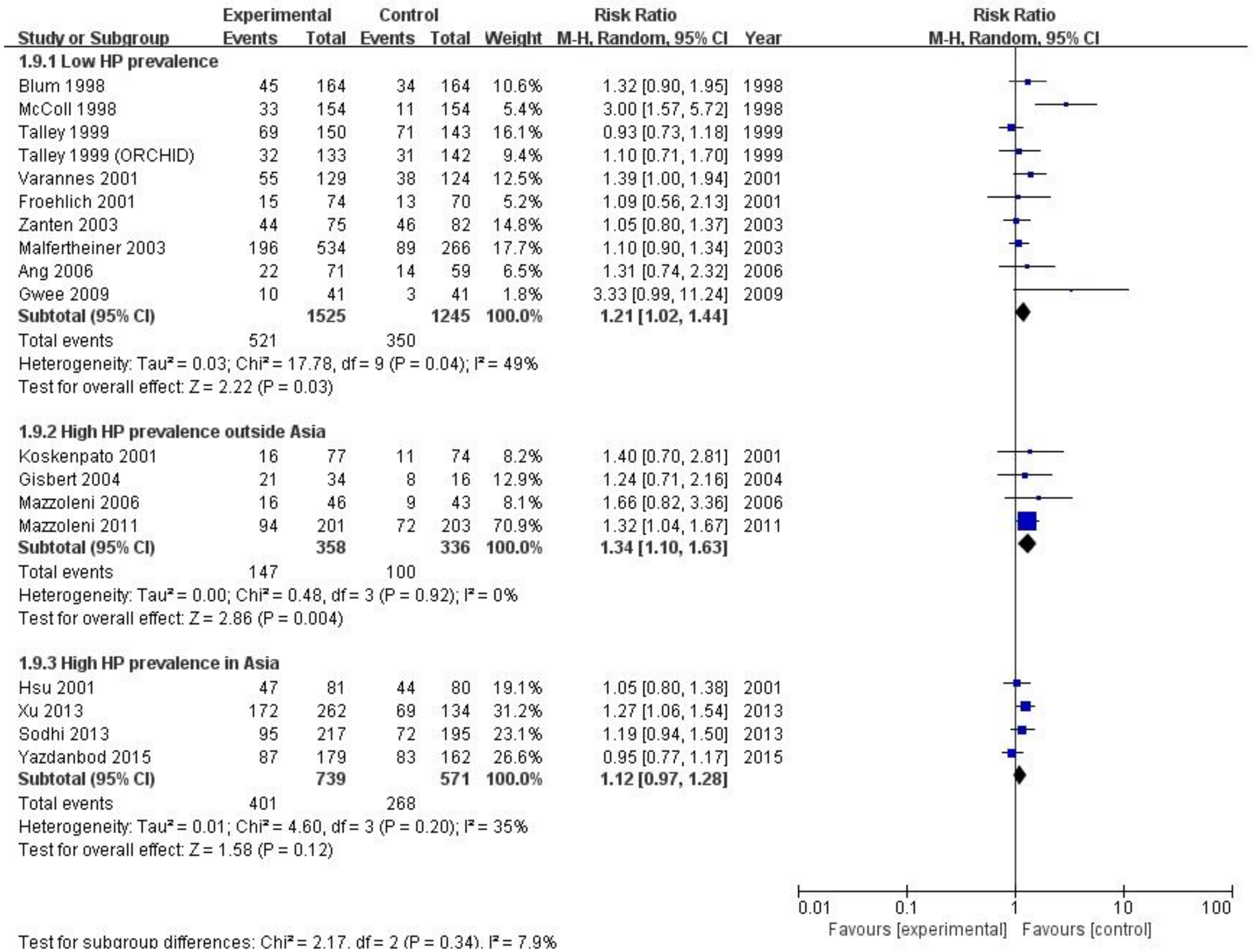

Test for subgroup differences: Chi² = 2.17, df = 2 (P = 0.34), I² = 7.9%

Figure S4. Subgroup analysis by *H. pylori* prevalence and geographical regions. 1.9.1 Low-prevalence region, 1.9.2 High-prevalence region outside Asia, 1.9.3 High-prevalence region in Asia. CI: confidence interval.
